# Terrestrial Adaptation in *Chelonoidis vicina* as Revealed Based on Analysis of the Complete Mitochondrial Genome

**DOI:** 10.3390/genes16020173

**Published:** 2025-01-30

**Authors:** Yao Chen, Xibao Wang, Xiaoyang Wu, Yongquan Shang, Qinguo Wei, Haotian Cai, Weilai Sha, Yan Qi, Shuli Liu, Honghai Zhang

**Affiliations:** 1 College of Life Sciences, Qufu Normal University, Qufu 273165, China; chenyao@qfnu.edu.cn (Y.C.); wangxibao1995@163.com (X.W.); wuxiaoyang1988@126.com (X.W.); yongquanshang@163.com (Y.S.); qgwei2008@163.com (Q.W.); caihaotian0915@163.com (H.C.); shaweilai@163.com (W.S.); qiyan94163@126.com (Y.Q.); 2 Zhonghuan Shengda Environmental Technology Group (Qingyun) Co., Ltd., Dezhou 253000, China; 13910335155@163.com

**Keywords:** *Chelonoidis vicina*, mitochondrial genomes, positive selection, evolution, adaptation

## Abstract

Background/Objectives: Mitochondrial genomes are widely used in phylogenetics and evolutionary and ecological research. Methods: In this study, the newest mitochondrial genome of *Chelonoidis vicina* was assembled and annotated. The comparative mitochondrial genome and selection pressure analyses were used to examine the terrestrial adaptive evolution characteristics of *C. vicina* and other terrestrial reptiles. Results: The results reveal that the mitochondrial genome of the tortoise *C. vicina* is consistent with that of other tortoise species, comprising 13 protein-coding genes (PCGs), 2 rRNAs, 22 tRNAs, and 1 noncoding control region (CR). The analysis of selection pressure reveals the presence of positive selection sites in the *COX2*, *COX3*, *Cytb*, *ND3*, *ND4*, *ND4L*, *ND5*, and *ND6* genes of terrestrial reptiles. Of these, the *COX2* and *ND3* genes exhibited faster evolutionary rates. The mitochondrial genome structure of *C. vicina* is consistent with that of different terrestrial reptiles. The positive selection sites of *COX2* and *ND3* in terrestrial reptiles are closely related to a change in mitochondrial energy metabolism, which is possibly related to terrestrial adaptability. Conclusions: The results of this study provide new insights into the adaptive evolution of *C. vicina* to terrestrial niches from a mitogenomic perspective, as well as genetic resources for the protection of *C. vicina*.

## Simple Summary

The mitochondrial genome has been widely used in biological phylogeny and evolutionary ecology due to its specific energy metabolism center and matrilineal inheritance. In this study, the mitochondrial genome of *Chelonoidis vicina* was assembled and annotated, and comparative mitochondrial genome, phylogenetic, and selection pressure analyses were used to examine the structure, phylogenetic status, and terrestrial adaptive evolution characteristics of *C. vicina.* These analyses can aid our understanding of the history of this ancient tortoise’s adaptive evolution to a terrestrial environment from a molecular evolution standpoint.

## 1. Introduction

Mitochondria are organelles involved in the function of energy metabolism in eukaryotic cells and that harbor a genome independent of the nuclear genome [1,2]. In vertebrates, the mitochondrial genome is a double-stranded closed-loop molecular structure of about 14 to 16 Kb [3]. The mitochondrial genome generally consists of two chains, a guanine-rich heavy (H) chain and a cytosine-rich light (L) chain, comprising four components, namely a control region (D-loop), 2 ribosomal RNA genes (rRNAs), 13 protein-coding genes (PCGs), and 22 transfer RNA genes (tRNAs) [3,4,5,6,7,8,9]. Compared with nuclear genomes, mitochondrial genomes have unique properties, such as regarding their size, the complete absence or lower frequency of recombination, matrilineal inheritance, and a highly conservative gene content and evolutionary rate [10,11,12]; therefore, the mitochondrial genome is an extremely useful resource for investigating vertebrate phylogeny, genetic diversity, and adaptive evolution [13,14,15]. A large number of studies have shown that in vertebrates, the evolution of their mitochondrial genome is related to their niche adaptation. For example, as members of the Mustelidae have adapted to different niches, their mitochondrial genomes have undergone different evolutionary processes, and similar evolutionary patterns are found in Cetartiodactyla mitochondrial genomes [16,17]. As another example, Vesicomyidae species have adapted to a deep-sea environment, and their mitochondrial genome shows evidence of positive selection signals [18]. In addition, the patterns of less stringent selection in the mitochondrial genomes of domestic animals (such as dogs, cattle, and yaks) are evidence of their adaptation to domesticated niches [19,20,21]. The mitochondrial genome of Tibetan loaches contains more nonsynonymous mutations for adapting to the Tibetan plateau [22]. Furthermore, in order to adapt to their habitat environment, the adaptive evolutionary attributes of several aquatic reptiles such as *Killifish* (*Fundulus* genus), the intertidal spider (*Desis jiaxiangi*), *Rhinogobius shennongensis*, *Rhinogobius wuyanlingensis*, and *Chaenogobius annularis* are characterized by a low mutation rate and positive selection in their PCGs [23,24,25]. In summary, research into the biogeography, population genetics, and adaptive evolution of a species is strengthened by the results of investigations into the mitochondrial genome.

*C. vicina* (also referred to as the Cerro Azul giant tortoise, the Iguana Cove tortoise, or the Isabela Island giant tortoise), belonging to Reptilia, Testudines, Testudinidae, and *Chelonoidis*, is a species of Galápagos tortoise endemic to the Galápagos Isabela Island [26]. The habitat of *C. vicina* has been destroyed by extensive overexploitation and the introduction of exotic species, resulting in their population decline [27,28,29,30,31]. In existing research on *C. vicina*, conservation biology researchers have mostly focused on large-scale ecological studies, including examining their biological characteristics, distribution scope, the causes of their endangered status, and conservation measures.

From a previous study [32], the entire mitochondrial genome of *C. vicina* has been assembled, which has already been used to resolve the phylogenetic history of giant tortoises across the Galapagos; however, the *ND2* coding sequences of *C. vicina* have several unrecognized base sequences (from 4199 bp to 4216 bp), which means the mitochondrial genome data cannot be used for the analysis of selection pressure. Therefore, in this study, the entire new mitochondrial genome of *C. vicina* was assembled and annotated using high-quality original genome sequencing data for evolutionary analysis. The aims of this study were to (1) investigate the structural properties of the *C. vicina* mitochondrial genome and (2) use bioinformatics and evolutionary analysis to investigate the evolutionary traits of the mitochondrial genome related to terrestrial habitat adaptation by comparison with other available mitochondrial genomes. And our results not only allow elucidating the most recent molecular processes in the adaptive evolution of terrestrial reptiles to a terrestrial environment but also provide genetic resources for the protection of *C. vicina*.

## 2. Materials and Methods

### 2.1. Complete Mitogenome Data and Species Sample

Using the GenBank database (http://www.ncbi.nlm.nih.gov/, accessed on 12 October 2024), the complete mitogenomes of 48 vertebrate species each with two different habitats (32 terrestrial reptiles and 16 aquatic reptiles) were downloaded to be used for comparisons in this study (Table S1).

Furthermore, high-quality raw genome sequencing data of *C. vicina* (SRX12027758) were downloaded from the SRA database (https://www.ncbi.nlm.nih.gov/sra/, accessed on 10 February 2022). The raw data were used to assemble the complete mitogenome using NOVOPlasty (V 4.1). The sequencing data were assembled using seed sequence data (accession number: NC_080265.1). The revised mitochondrial genome sequences were annotated using MITOS2 of Galaxy tools, a free online platform for data analysis (https://usegalaxy.eu/, accessed on 18 October 2024) [33]. After annotation, the results were compared with the mitogenome of *C. carbonarius* (accession number: NC_080265.1) using BLAST searches.

### 2.2. Mitogenome Analyses

The structural map of the *C. vicina* mitogenome was investigated using OGDRAW (V 1.3.1, https://chlorobox.mpimp-golm.mpg.de/OGDraw.html, accessed on 23 October 2024) [34]. Then, relative synonymous codon usage (RSCU) of the *C. vicina* mitogenome was calculated using MEGA X [35] and presented using R software (V 4.1.3; package, ggplot2). The tRNA gene structures of the *C. vicina* mitogenome were predicted using tRNAScan-SE (V 2.0) [36]. The effective number of codons (Enc) and the GC content of the third codon positions (GC3s) were determined using Codon W (V 1.4.4) [37]. The composition skew values were calculated according to the following formulas: AT skew [(A *−* T)/(A + T)] and GC skew [(G *−* C)/(G + C)] [38]. The rose plot for amino acid usage in the *C*. *vicina* mitogenome is represented as heatmaps, created using ggplot2 of R software (V 4.1.3).

### 2.3. Comparative Mitochondrial Genome Analyses

The AT and GC skew values were used to measure nucleotide compositional bias [38]. Additionally, a synteny analysis was conducted among 14 mitochondrial genomes, selected from the abovementioned 48 species and *C*. *vicina*, using Mauve software (V 2.4.0) [39]. Comparative codon and amino acid usage among the 48 selected mitogenomes and *C*. *vicina* are represented as heatmaps, which were generated using ggplot2 of R software (V 4.1.3).

### 2.4. Phylogenetic Construction

In order to determine the phylogenetic status of *C. vicina*, we selected the sequences of 48 mitogenomes (Table S1) for concatenation in representing the 13 different characteristic protein-coding genes (PCGs). The *Gallus gallus* was selected as the outgroup. The 13 mtDNA PCGs were aligned by using MUSCLE (V 3.8.31) [40]. For BI analysis, the GTR + I + G model was constructed from the BIC scores in ModelFinder to perform Bayesian inference (BI) analysis by following previous research [41]. BI analysis was determined using Mr. Bayes software (V 3.2.6) [42] under following four independent chains running for 100,000 generations, sub-sampling every 1000 generations, and using a burn-in of 100 generations. MEGA X [35] was used to edit the resulting phylogenetic trees. Finally, the BI phylogenetic tree was edited and visualized using online tools at the Interactive Tree of Life (ITOL) website (https://itol.embl.de/, accessed on 25 October 2024) [43].

### 2.5. Selection Pressure Analyses

The *ω* values were 1, <1, or >1, indicating neutral mutation, negative (purifying) selection, or positive (diversifying) selection, respectively. In our study, the branch model (one-ratio (M0) model, two-ratio (M2) model, and NSsites = 0) was selected to detect each mtDNA PCG in the 49 species. The terrestrial reptiles (TRs) were set as foreground branches, and the aquatic reptiles (ARs) were set as the background branches. Furthermore, the branch-site model (one-ratio (M0) model, two-ratio (M2) model, and NSsites = 2) from PAML (V 4.3) [44] was used to detect each mtDNA PCG of the 49 species (foreground branches: TRs; background branches: ARs). The *p*-values were corrected using the false discovery rate (FDR) [23]. The *p*-value of LRTs was used to identify the genes of mtDNA PCGs that had undergone rapid evolution. All analyses of the selection pressure were based on the BI tree.

## 3. Result

### 3.1. Mitogenome Organization and Structure

**Figure 1:**
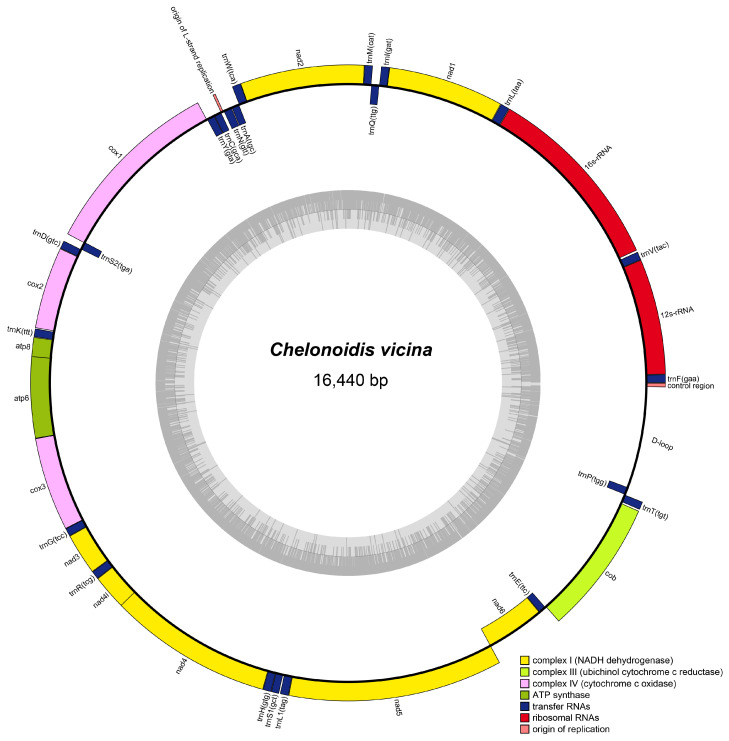
Map of *C*. *vicina* mitogenome genes plotted on the outer circle are encoded on the heavy strand (H-strand) and indicated by clockwise arrows, while genes plotted on the inner circle are encoded on the light strand (L-strand) and indicated by counterclockwise arrows.

In this study, the complete mitogenome of the Cerro Azul giant tortoise (*C. vicina*) was assembled and uploaded to GenBank with the accession number PP475397. The mitogenome of *C. vicina* is 16,440 bp and includes 13 protein-coding genes (PCGs), 2 ribosomal RNA genes (12S rRNA and 16S rRNA), 1 control region (D-loop), and 22 transfer RNA genes (tRNA). There are eight tRNAs (*tRNA^Gln^*, *tRNAAla*, t*RNAAsn*, *tRNACys, tRNATy*^r^, *tRNASer*, *tRNAGlu*, and *tRNAPro*) and one PCG (*ND6*) located on the light (L or −) strand, while the others are located on the heavy (H or +) strand (Figure 1).

The total sequence length of the PCGs is 11,370 bp, of which the longest gene is *ND5* (1797 bp), while the shortest genes are *tRNA^Cys^* (66 bp) and *tRNA^Ser^* (66 bp), which are, respectively, located in the heavy and light strands (Table 1). Gene spacing or gene overlap is observed between adjacent genes in the *C. vicina* mitogenome. There are 16 gene spacers with a total of 75 bp, of which the longest interval is 26 bp between *tRNA^Cys^* and *tRNA^Tyr^*. And there are nine gene overlaps, with a total length of 29 bp. The longest overlap, between *ATP8* and *ATP6*, is 10 bp long (Table 1). ATG is the most frequently used start codon for the eight protein-coding genes, with *COX1* being the exception in using the GTG codon for initiation (Table 1).

We analyzed the mitogenome compositions of *C*. *vicina*, which showed a distinct AT bias (Table 2). The nucleotide composition of the total *C*. *vicina* mitogenome is adenine (A)-5726 (34.83%), thymine (T)-4044 (24.60%), guanine (G)-2067 (12.57%), and cytosine (C)-4603 (28.00%). The proportion of AT (59.43%) was higher than that of GC (40.57%). In addition, the total AT and GC skew is 0.85 and −1.53, respectively (Table 2), indicating a higher content of A than T nucleotides and a higher content of C than G nucleotides, respectively.

### 3.2. Protein-Coding Genes

**Figure 2:**
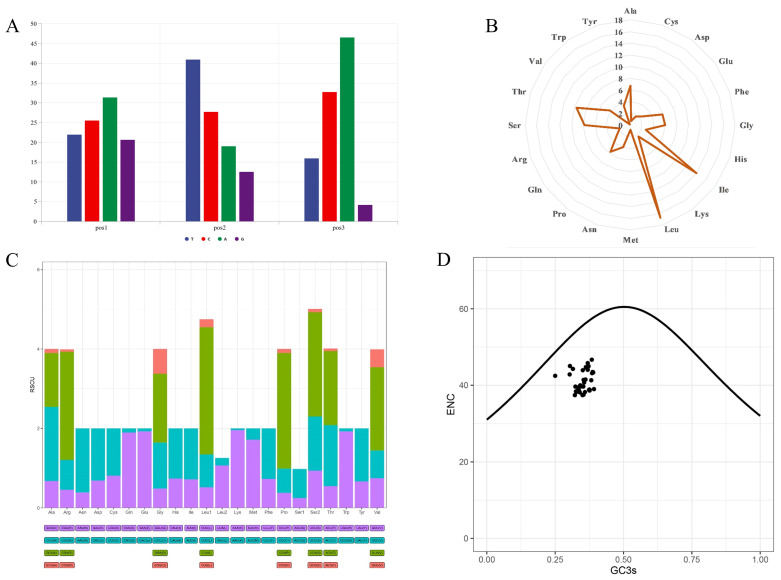
(**A**) Position-specific nucleotide usage in the *C*. *vicina* mitogenome. (**B**) Rose plot of amino acid usage in the *C*. *vicina* mitogenome. (**C**) Relative synonymous codon usage (RSCU) in *C*. *vicina*. In the *X*-axis, each amino acid encoded by different codon families is represented using different colors; the *Y*-axis represents the RSCU values. (**D**) Plot of effective number of codons (ENc) of 49 mitogenomes below the curve of selection pressure.

The entire length of the PCGs is 11,340 bp, which accounts for 68.98% of the *C*. *vicina* mitogenome (Table 2). The occurrence of A and C in the third codon position was found to be 46.61% and 32.80%, respectively, based on analysis of the position-specific nucleotide usage (Figure 2A), which was validated by analysis of the relative synonymous codon usage (RSCU) (Figure 2C). Additionally, according to the RSCU, the codons CUA (327, 8.65%), AUC (207, 5.48%), and AUA (194, 5.13%) were the most prevalent, whereas CGG (1, 0.03%), AAG (2, 0.05%), and GAG (3, 0.08%) were the least prevalent (Figure 2C). The total length of the translated PCGs is 3780 amino acids. In the *C*. *vicina* mitogenome, translation of 12 of the PCGs was found to be initiated by an ATG start codon, namely, *ND1*, *ND2*, *COX2*, *ATP8*, *ATP6*, *COX3*, *nad3*, *ND4L*, *ND4*, *ND5*, *ND6*, and *Cytb*, while *COX1* is the exception and uses a GTG codon for initiation (Table 2). TAA is the most frequent stop codon, found in the sequences of *COX2*, *ATP8*, *ATP6*, *ND4L*, and *ND5*. The PCGs of *ND1* and *ND2* have TAG as their stop codon, while AGG was only associated with *COX1*. The stop codons of the PCGs *Cytb*, *COX3*, *nad3*, and *ND4* are incomplete, with TA, TA, T, and T, respectively. In addition, from the analysis of amino acid usage in the *C. vicina* genome, we could determine that leucine (L, 485, 12.83%), threonine (T, 356, 9.42%), and isoleucine (I, 323, 8.54%) are the most preferred, while cysteine (C, 27, 0.714%), aspartate (D, 64, 0.17%), and arginine (R, 69, 0.18%) are the least preferred (Figure 2B). Furthermore, analysis of the effective number of codons (ENc) revealed that all the 49 mitogenomes investigated in this study were below the selection pressure curve, which indicates that there is natural selection acting on all these mitogenomes (Figure 2D).

### 3.3. RNA Genes in C. vicina Mitogenomes

**Figure 3:**
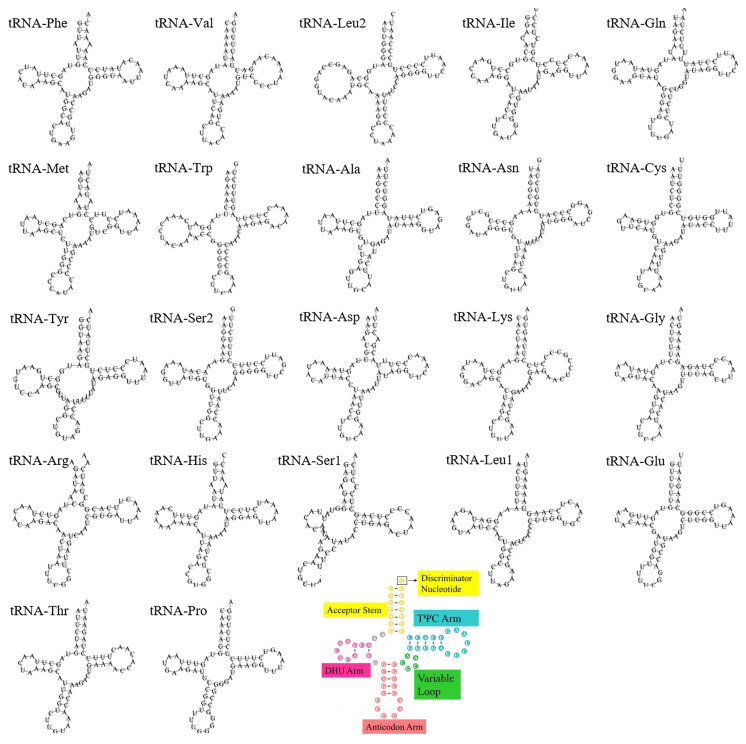
The secondary structures of 22 tRNAs in the *C. vicina* mitogenome. Note: the last colored tRNA secondary structures have a canonical clover leaf structure.

There are two ribosomal RNAs (rRNAs) in the *C. vicina* mitogenomes, in which the content of A+T accounted for 58.44% (Table 1). In addition, the *12S rRNA* and *16S rRNA* genes were identified with lengths of 969 bp and 1593 bp, respectively (Table 2), and an AT and GC skew of 0.17 and −0.22, respectively (Table 2). In addition, there are 22 transfer RNAs (tRNAs) in the mitogenome of *C. vicina*, in which the content of A+T accounted for 61.49% (Table 3). The lengths of the tRNAs are 66–76 bp with a 0.31 AT skew and −0.19 GC skew, which indicates that there is a higher content of A (38.37%) than of T (20.07%) and higher content of C (24.82%) than of G (16.74%) (Table 3). In total, 21 tRNAs have a canonical clover leaf structure. All of them lack a variable loop while *tRNA^Ser1^*(GCT) lacks a dihydrouridine hairpin structure (Figure 3).

### 3.4. Comparing Mitogenomes Among Species

The nucleotide composition of mitogenomes was generally consistent between the 49 species. In addition, the A% (32.4%) was higher than the T% (26.4%), while the G% (12.5%) was lower than the C% (28.7%). There was a positive AT skew and negative GC skew. In addition, the AT skew differs with a positive value greatly by 49 species whereas the GC skew of each species does not differ much with a negative value. And it should be noted that the AT skew values of the same group do not differ much, e.g., *Naja*, *Chelonoidis,* and *Testudo*.

**Figure 4:**
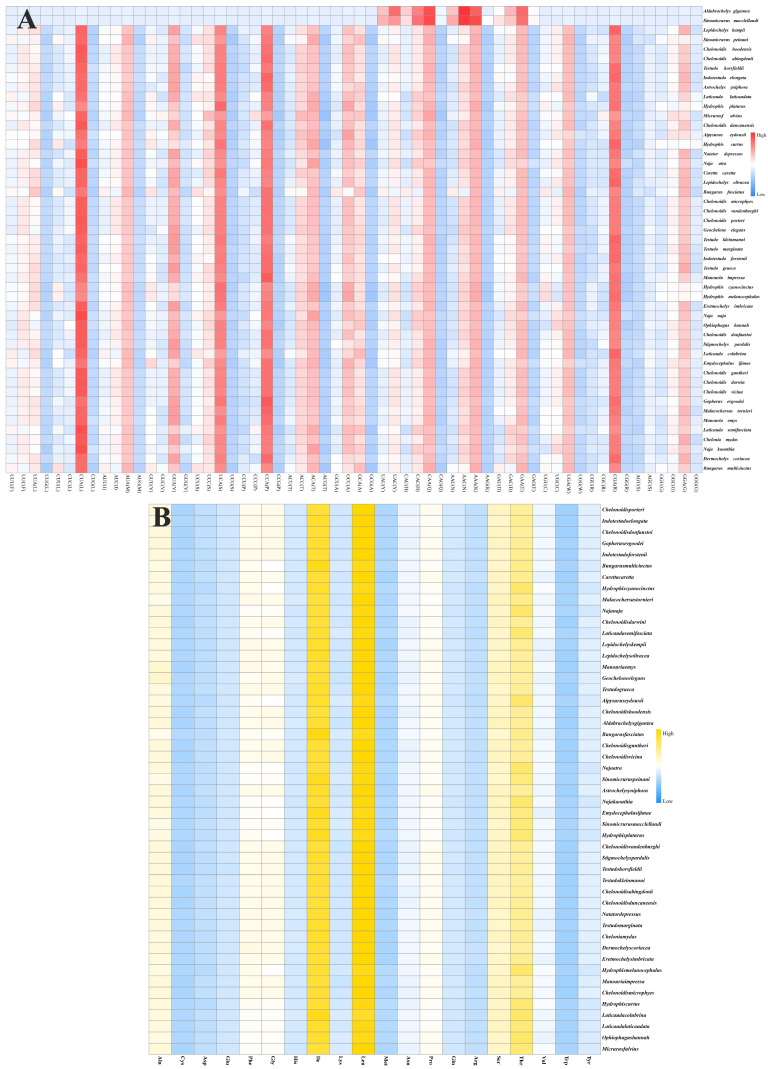
Heatmap of codon usage in the mitogenomes of the 49 species. Note: (**A**), heatmap of nucleic acid composition of 49 species; (**B**), heatmap of amino acid composition of 49 species; red represents the most used codons, yellow represents the most used amino acids, and blue represents rarely used codons.

**Figure 5:**
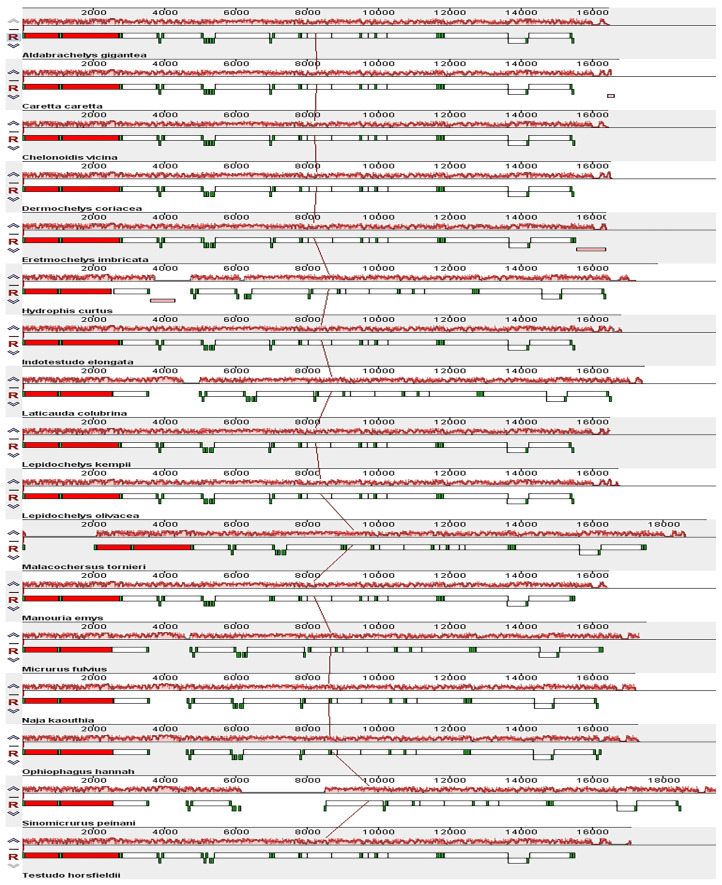
Collinearity analysis of the mitochondrial genome of 17 species analyzed in this study using Mauve. PCGs: white block; 12S rRNA and 16S rRNA: red block; tRNA: green block.

The heatmap of the codon usage in the mitogenomes of the 49 species shows that the codons CAA (Q), AAA (K), GAA (E), CUA (L), CCA (P), CGA (R), and UCA (S) were the most used (Figure 4A). In addition, the amino acids isoleucine (Ile, I), leucine (Leu, L), threonine (Thr, T), serine (Ser, S), alanine (Ala, A), and proline (Pro, P) were the most prevalent (Figure 4B). Comparative alignment of the 17 mitochondrial genomes showed that the gene order was the same in *C. vicina* and 16 other species investigated in this study (Figure 5).

### 3.5. Phylogenetic Analyses

**Figure 6:**
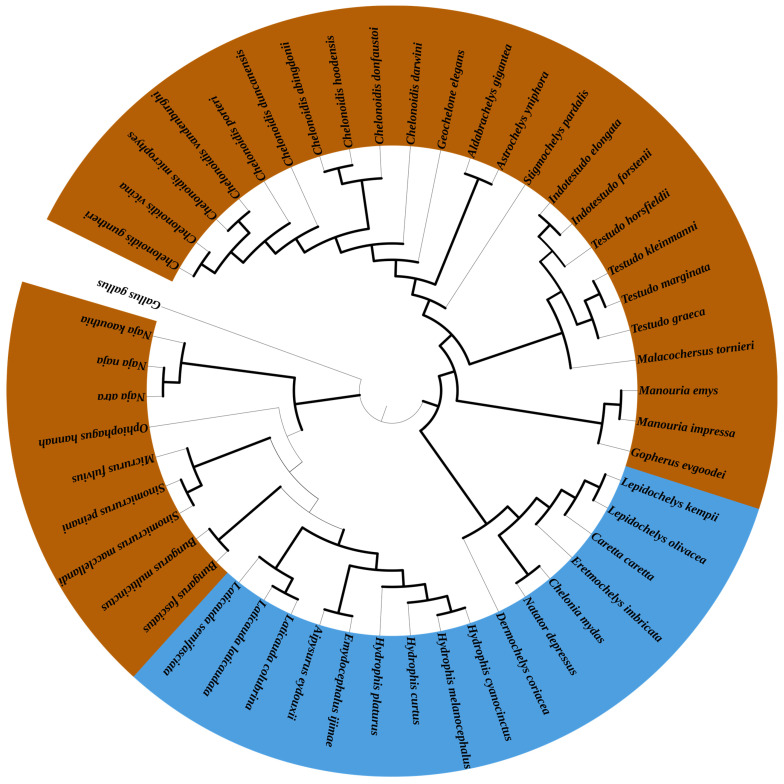
The phylogenetic relationships of 49 species based on BI tree using 13 concatenated mitochondrial PCGs. Note: *C. vicina* shared a closer common ancestor with *Chelonoidis guntheri*. The genus *Chelonoidis* was clustered into one clade that was closest to *Geochelone*. Among the sea snakes, three species of *Laticauda* and *Hydrophis* differentiated earlier and formed a separate branch. In addition, *Aipysurus* and *Emydocephalus* differentiated from *Hydrophis* to form a branch. Bayes software (V 3.2.6) under four simultaneous Markov chain Monte Carlo (MCMC) iterations, each of which was run for 2,000,000 cycles. Sampling was conducted every 1000 generations. Brown and blue represent terrestrial and aquatic reptiles, respectively. *G. gallus* was used as an outgroup.

The Bayesian (BI) phylogenetic tree revealed that there are two branches including Testudoformes and Serpentes within the 49 species (Figure 6). Subsequently, *C. vicina* shared a closer common ancestor with *Chelonoidis guntheri*. Species of the genus *Chelonoidis* were clustered into one clade that was closest to *Geochelone*. This also serves as support for the mitochondrial genome data of *C. vicina* being correctly assembled in our study. Among the sea snakes, three species of *Laticauda* and *Hydrophis* differentiated earlier and formed a separate branch. In addition, *Aipysurus* and *Emydocephalus* differentiated from *Hydrophis* to form a branch.

### 3.6. Evolutionary Analysis

The *dN*/*dS* values of 13 PCGs of the 49 species were estimated to evaluate the mutational pressure of the codons from purifying selection under the branch model within the PAML package. The evolutionary rates of the mtDNA PCGs in the 49 species investigated in this study differed under different environmental selection pressures. The branch-site model was used to detect the positive site of each mtDNA PCG in the 49 species. From the results of the analysis of the branch-site model (Table 4), we determined that there are six positively selected site genes *COX2* (*p* = 1; positively selected sites 5 and 16), *COX3* (*p* = 1; positively selected sites 22 and 175), *CYTB* (*p* = 1; positively selected site 324), *ND3* (*p* = 1; positively selected sites 14 and 16), *ND4* (*p* = 1; positively selected sites 30 and 431), *ND4L* (*p* = 1; positively selected sites 15 and 56), *ND5* (*p* = 1; positively selected site 535), and *ND6* (*p* = 1; positively selected site 130). By using the branch model, there are three genes that show significant differences, namely *COX2* (*p* = 0.012), *ND1* (*p* = 0.003), and *ND3* (*p* = 0.002). Meanwhile, *COX2* (*ω*_AR_ = 0.024; *ω*_TR_ = 0.039) and *ND3* (*ω*_AR_ = 0.060; *ω*_TR_ = 0.112) are two rapidly evolving genes within the 32 terrestrial reptiles, and *ND1* (*ω*_AR_ = 0.049; *ω*_TR_ = 0.035) is a rapidly evolving gene within the 16 aquatic reptiles (Table 5).

## 4. Discussion

Mitochondria, as energy-generating organelle, can provide the energy required for animals’ life activities through oxidative phosphorylation [45,46,47]. In evolutionary biology and conservation genetics, mitochondrial genomes are used to analyze phylogenetic relationships and adaptive evolution. Therefore, in this study, we assembled the mitochondrial genome of *C. vicina* and compared it with that of 32 terrestrial reptiles and 16 aquatic reptiles in order to clarify how terrestrial reptiles have adapted to their terrestrial niche, thereby providing a scientific and theoretical basis for their protection.

### 4.1. Mitochondrial Genome Characteristics

The characteristics of the mitochondrial genome of different animal groups may vary. The mitochondrial genome of terrestrial reptiles is usually a double-stranded circular molecule, about 15–20 Kb in size, and containing 37 genes, including 13 PCGs, 2 rRNAs, 22 tRNAs, and 1 control region. In most PCGs, the start codon is ATG and the stop codon is TAA [48]. Here, we assembled and annotated the mitochondrial genome of *C. vicina*, and the results (Figure 1 and Table 2) show that its characteristics are similar to those of other terrestrial reptiles. Previous studies have shown that in protozoa, the GC offset rate is indicative of a DNA replication-driven genome evolution event [49]. The *C. vicina* mitochondrial genome is characterized by a higher AT than GC content, similarly to other vertebrates [24]. In addition, in this study, the AT content was also found to be higher than the GC content for other terrestrial reptiles (Table 4), but it is not clear whether there is a relationship between the GC skewness and species evolution, especially in *C. vicina*. In terms of the secondary structure of *C. vicina* tRNAs, only *tRNA^SER2^* (GCT) lacks a dihydrouridine hairpin structure, and the other 21 tRNAs have a typical clover structure. There are some studies showing that *tRNA^SER^* lacks a typical clover structure in several animals [41,50], while others have shown that the lack of a dihydrouridine arm or thymidine–pseudo-uridine–cytidine (T-ψ-C) ring in *tRNA^SER^* may not affect its normal function [51].

### 4.2. Phylogenetic Analysis

In the phylogenetic analysis, all animals of Testudoformes were clustered into one branch, while animals of Serpentes were clustered into another branch, which validates the use of mitochondrial genomes in the systematic taxonomy classification of species. All the species of the genus *Chelonoidis* grouped into one branch, where *C. guntheri* and *C. vicina* are the most closely genetically related, which is consistent with previous research results on the traditional classification of *C. vicin* [32].

### 4.3. Evolutionary Analysis of Terrestrial Adaptability

The selection stress intensity of protein-coding genes is measured based on the nonsynonymous/synonymous nucleotide substitution ratio (i.e., the *dN*/*dS* ratio or *ω* value) [52,53]. In this study, the result of the ENc plot revealed that all the 49 species bore natural selection (Figure 2D); therefore, it is necessary to conduct selection stress analysis to screen positive selection genes. After the analysis, the *ω* value of each mtDNA PCG of the 49 species was generally less than 1, indicating that the PCG of vertebrate mitochondrial DNA is in a state of purifying selection in different habitats. In addition, we used the branch-site model to detect positively selected genes. The results showed that compared with aquatic reptiles, the *COX2*, *COX3*, *Cytb*, *ND3*, *ND4*, *ND4L*, *ND5*, and *ND6* genes of terrestrial reptiles contain unique positive selection sites (Table 5). Of these, the *COX2* and *ND3* genes not only have positive selection sites but also have faster evolutionary rates in terrestrial reptiles than in aquatic reptiles (Table 5). This suggests that tortoises accumulate more non-identical mutations in these three genes. Studies have shown that a large number of nonsynonymous mutations may lead to some useful changes in amino acids related to defense [22,23,54]. In fact, the proteins encoded by *ND* genes are concentrated mostly in mitochondrial complex I to respond to various stresses [55]. Mitochondrial complex I is a large enzyme that can affect mitochondrial differential reactive oxygen species (ROS) through the electronic respiratory chain. And ROS is closely related to energy production [56]. In addition, the *COX* genes are located in cytochrome c oxidase (Complex IV), which is the last and rate-limiting step in the electronic respiratory chain and closely related to the prevention of the formation of ROS [57]. Therefore, the positive selection sites of *COX2* and *ND3* in terrestrial reptiles are closely related to a change in mitochondrial energy metabolism, which is possibly related to terrestrial adaptability.

## 5. Conclusions

In this study, the high-quality mitochondrial genomes of *C. vicina* were constructed, and comparative mitogenomic analyses of 32 terrestrial reptiles and 16 aquatic reptiles were conducted. We (1) found the structural characteristics of the mitochondrial genome of the tortoise *C. vicina* are consistent with those of other tortoise species; (2) the AT skew differs with a positive value greatly by 49 species whereas the GC skew of each species does not differ much with a negative value; (3) *C. vicina* shared a closer common ancestor with *Chelonoidis guntheri*. The genus *Chelonoidis* was clustered into one clade that was closest to *Geochelone*; (4) *COX2* and *ND3* are two rapidly evolving genes within the 32 terrestrial reptiles; meanwhile, *ND1* is a rapidly evolving gene within the 16 aquatic reptiles; and (5) the positive selection sites of *COX2* and *ND3* in terrestrial reptiles are closely related to a change in mitochondrial energy metabolism, which is possibly related to terrestrial adaptability. Overall, this study is a blueprint for further research on the protection of *C. vicina* based on the comparative mitogenomic analyses and evolutionary analyses and which provide important basic data for the future research and conservation of *Chelonoidis*.

## Figures and Tables

**Table 1:** Characteristics of the mitochondrial genome of *C. vicina*.

Gene	Start	Stop	Strand	Length	Intergenic Nucleotide	Star	Stop
*tRNA^Phe^*	1	70	+	70	0		
*12s rRNA*	71	1039	+	969	0		
*tRNA^Val^*	1040	1110	+	71	14		
*16s rRNA*	1125	2717	+	1593	1		
*tRNA^Leu^*	2719	2794	+	76	1		
*ND1*	2796	3767	+	972	−1	ATG	TAG
*tRNA^Ile^*	3767	3836	+	70	−1		
*tRNA^Gln^*	3836	3906	−	71	−1		
*tRNA^Met^*	3906	3974	+	69	0		
*ND2*	3975	5015	+	1041	−2	ATG	TAG
*tRNA^Trp^*	5014	5088	+	75	1		
*tRNA^Ala^*	5090	5158	−	69	2		
*tRNA^Asn^*	5161	5234	−	74	3		
*tRNA^Cys^*	5261	5326	−	66	26		
*tRNA^Tyr^*	5327	5397	−	71	1		
*COX1*	5399	6946	+	1548	3	GTG	AGG
*tRNA^Ser^*	6938	7008	−	71	0		
*tRNA^Asp^*	7009	7078	+	70	0		
*COX2*	7079	7765	+	687	5	ATG	TAA
*tRNA^Lys^*	7771	7840	+	70	1		
*ATP8*	7842	8006	+	165	−10	ATG	TAA
*ATP6*	7997	8680	+	684	−1	ATG	TAA
*COX3*	8680	9464	+	785	−1	ATG	TA
*tRNA^Gly^*	9464	9531	+	68	0		
*ND3*	9532	9883	+	180	0	ATG	TA
*tRNA^Arg^*	9882	9951	+	70	1		
*ND4L*	9952	10,248	+	297	0	ATG	TAA
*ND4*	10,242	11,619	+	1378	−7	ATG	T
*tRNA^His^*	11,620	11,689	+	70	0		
*tRNA^Ser^*	11,690	11,755	+	66	0		
*tRNA^Leu^*	11,765	11,836	+	72	9		
*ND5*	11,837	13,633	+	1797	0	ATG	TAA
*ND6*	13,629	14,153	−	525	−5	ATG	AGG
*tRNA^Glu^*	14,154	14,221	−	68	0		
*Cytb*	14,226	15,357	+	1132	4	ATG	TA
*tRNA^Thr^*	15,370	15,438	+	69	2		
*tRNA^Pro^*	15,440	15,508	−	69	1		

**Table 2:** Nucleotide composition and AT and GC skew in *C. vicina*.

*C. vicina*	Size	A %	T %	G %	C %	AT %	GC %	AT Skew	GC Skew
mtDNA	16,440	34.83	24.60	12.57	28.00	59.43	40.57	0.17	−0.38
PCGs	11,340	34.02	24.87	11.34	29.77	58.89	41.11	0.16	−0.45
tRNAs	1545	35.86	25.63	15.08	23.43	61.49	38.51	0.17	−0.22
rRNAs	2562	38.37	20.07	16.74	24.82	58.44	41.56	0.31	−0.19
D-Loop	932	34.44	31.97	11.92	21.67	66.41	33.59	0.04	−0.29

**Table 3:** The mitogenome composition of 49 species.

Spcies	T(U)%	A%	AT%	AT Skew	G%	C%	GC%	GC Skew
*Bungarus fasciatus*	31.3	33.5	64.8	0.034	11.2	24	35.2	−0.362
*Bungarus multicinctus*	29.9	32.2	62.1	0.037	12.1	25.8	37.9	−0.362
*Micrurus fulvius*	27.7	30.8	58.5	0.053	13.0	28.5	41.5	−0.373
*Naja naja*	26.2	32.1	58.3	0.102	13.2	28.5	41.7	−0.367
*Naja kaouthia*	26.2	32.2	58.3	0.103	13.2	28.5	41.7	−0.368
*Naja atra*	26.2	32.2	58.4	0.101	13.1	28.5	41.6	−0.371
*Ophiophagus hannah*	26.1	32.7	58.8	0.113	12.1	29.1	41.2	−0.413
*Sinomicrurus peinani*	29.3	32.4	61.7	0.051	11.8	26.5	38.3	−0.383
*Sinomicrurus macclellandi*	27.7	32.0	59.6	0.072	12.2	28.2	40.4	−0.397
*Aldabrachelys gigantea*	26.4	32.8	59.2	0.107	12.4	28.4	40.8	−0.392
*Chelonoidis microphyes*	26.4	32.3	58.8	0.100	12.5	28.7	41.2	−0.394
*Chelonoidis vandenburghi*	26.4	32.4	58.8	0.100	12.4	28.7	41.2	−0.396
*Chelonoidis guntheri*	26.4	32.4	58.8	0.109	12.4	28.7	41.2	−0.396
*Chelonoidis donfaustoi*	26.4	32.4	58.8	0.102	12.4	28.8	41.2	−0.397
*Chelonoidis darwini*	26.4	32.5	58.8	0.104	12.4	28.8	41.2	−0.399
*C. vicina*	26.4	32.4	58.8	0.108	12.5	28.7	41.2	−0.395
*Chelonoidis porteri*	26.4	32.4	58.8	0.108	12.4	28.8	41.2	−0.399
*Chelonoidis duncanensis*	26.3	32.5	58.8	0.104	12.4	28.8	41.2	−0.399
*Chelonoidis hoodensis*	26.4	32.4	58.8	0.102	12.4	28.8	41.2	−0.397
*Chelonoidis abingdonii*	26.4	32.4	58.8	0.102	12.4	28.8	41.2	−0.397
*Gopherus evgoodei*	27.4	32.3	59.7	0.082	12.9	27.4	40.3	−0.358
*Geochelone elegans*	25.6	32.0	57.6	0.112	13.2	29.3	42.4	−0.380
*Malacochersus tornieri*	27.7	33.0	60.7	0.087	11.8	27.5	39.3	−0.399
*Testudo kleinmanni*	27.9	32.3	60.2	0.073	12.5	27.2	39.8	−0.370
*Testudo marginata*	27.8	32.3	60.1	0.076	12.7	27.3	39.9	−0.366
*Testudo horsfieldii*	27.5	32	59.5	0.077	12.8	27.7	40.5	−0.369
*Indotestudo forstenii*	27.8	32.8	60.7	0.082	12.1	27.2	39.3	−0.382
*Stigmochelys pardalis*	27.5	32.5	60.1	0.084	12.6	27.4	39.9	−0.371
*Manouria emys*	27.5	31.7	59.2	0.069	13.3	27.5	40.8	−0.350
*Testudo graeca*	28.3	32.9	61.2	0.074	12.2	26.6	38.8	−0.372
*Indotestudo elongata*	28.1	33.0	61.1	0.079	12.0	26.9	38.9	−0.384
*Manouria impressa*	28.0	31.6	59.6	0.059	13.3	27.1	40.4	−0.341
*Astrochelys yniphora*	27.5	32.5	60.1	0.083	12.5	27.4	39.9	−0.371
*Laticauda semifasciata*	27.3	31.9	59.2	0.079	13.2	27.6	40.8	−0.352
*Laticauda colubrina*	29.1	33.1	62.2	0.065	12.1	25.7	37.8	−0.360
*Laticauda laticaudata*	28.0	32.1	60.2	0.068	12.8	27.1	39.8	−0.358
*Aipysurus eydouxii*	26.8	31.9	58.7	0.086	12.7	28.6	41.3	−0.384
*Emydocephalus ijimae*	28.0	31.9	59.9	0.066	12.6	27.6	40.1	−0.373
*Hydrophis curtus*	28.3	31.0	59.3	0.046	13.5	27.2	40.7	−0.337
*Hydrophis cyanocinctus*	28.2	31.4	59.6	0.054	13.0	27.3	40.4	−0.354

**Table 4:** Positive selection of eight mtDNA PCGs using the branch-site model.

Gene	Model	2Δlnl	*p*-Value	Positively Selected Sites (BEB Analysis)
*COX2*	Model A vs. Null Mode	0	1	5 T 0.986 *, 16 T 0.985 *
*COX3*		0	1	22 M 0.955 *, 175 A 0.962 *
*CYtb*		0	1	324 T 0.994 **
*ND3*		0	1	14 S 0.998 **, 16 L 0.975 *
*ND4*		0	1	30 Y 0.975 *, 431 I 0.994**
*ND4L*		0	1	15 T 0.990 *,56 Q 0.962 *
*ND5*		0	1	535 S 0.970 *
*ND6*		0	1	130 G 0.977 *

Note: T, threonine, Thr; M, methionine, Met; A, alanine, Ala; S, serine, Ser; L, leucine, Leu; Y, tyrosine, Tyr; I, isoleucine, Ile; Q, glutarnine, Gln; G, Glycine, Gly; *, have significance, **, have very significance.

**Table 5:** Test for positive selection in divergent clades of each mtDNA PCG using branch model.

Gene	Model Compared	|2ΔlnL|	*p*-Value	*ω*_AR_	*ω*_TR_
*ATP6*	M2 vs. M0	0.635	0.426	0.095	0.085
*ATP8*		0.049	0.825	0.209	0.198
*COX1*		0.518	0.472	0.017	0.015
*COX2*		6.322	0.012 *	0.024	0.039
*COX3*		0.021	0.885	0.033	0.034
*Cytb*		0.087	0.768	0.044	0.046
*ND1*		8.989	0.003 **	0.049	0.035
*ND2*		0.133	0.715	0.067	0.064
*ND3*		10.256	0.002 **	0.060	0.112
*ND4*		3.462	0.063	0.061	0.050
*ND4L*		0.017	0.898	0.057	0.055
*ND5*		0.609	0.435	0.071	0.066
*ND6*		2.074	0.150	0.090	0.116

*, have significance, **, have very significance.

## Data Availability

The mitochondrial genome of *C. vicina* is available at GenBank repository (https://www.ncbi.nlm.nih.gov/, accessed on 12 October 2024), with the accession number: PP475397. The complete mitogenome information for 49 reptiles is available in Table S1.
